# Therapeutic Effects of Combined Treatment with the AEA Hydrolysis Inhibitor PF04457845 and the Substrate Selective COX-2 Inhibitor LM4131 in the Mouse Model of Neuropathic Pain

**DOI:** 10.3390/cells12091275

**Published:** 2023-04-27

**Authors:** Jie Wen, Scott Sackett, Mikiei Tanaka, Yumin Zhang

**Affiliations:** Department of Anatomy, Physiology and Genetics, Uniformed Services University of the Health Sciences, 4301 Jones Bridge Road, Bethesda, MD 20814, USA; jie.wen.ctr@usuhs.edu (J.W.); scottjsackett@gmail.com (S.S.); mikiei.tanaka.ctr@usuhs.edu (M.T.)

**Keywords:** anandamide, fatty acid amide hydrolase, substrate-selective COX-2 inhibitor, inflammation, neuropathic pain

## Abstract

Chronic neuropathic pain resulting from peripheral nerve damage is a significant clinical problem, which makes it imperative to develop the mechanism-based therapeutic approaches. Enhancement of endogenous cannabinoids by blocking their hydrolysis has been shown to reduce inflammation and neuronal damage in a number of neurological disorders and neurodegenerative diseases. However, recent studies suggest that inhibition of their hydrolysis can shift endocannabinoids 2-arachidonoyl glycerol (2-AG) and anandamide (AEA) toward the oxygenation pathway mediated by cyclooxygenase-2 (COX-2) to produce proinflammatory prostaglandin glycerol esters (PG-Gs) and prostaglandin ethanolamides (PG-EAs). Thus, blocking both endocannabinoid hydrolysis and oxygenation is likely to be more clinically beneficial. In this study, we used the chronic constriction injury (CCI) mouse model to explore the therapeutic effects of simultaneous inhibition of AEA hydrolysis and oxygenation in the treatment of neuropathic pain. We found that the fatty acid amide hydrolase (FAAH) inhibitor PF04457845 and the substrate-selective COX-2 inhibitor LM4131 dose-dependently reduced thermal hyperalgesia and mechanical allodynia in the CCI mice. In addition to ameliorating the pain behaviors, combined treatment with subeffective doses of these inhibitors greatly attenuated the accumulation of inflammatory cells in both sciatic nerve and spinal cord. Consistently, the increased proinflammatory cytokines IL-1β, IL-6, and chemokine MCP-1 in the CCI mouse spinal cord and sciatic nerve were also significantly reduced by combination of low doses of PF04457845 and LM4131 treatment. Therefore, our study suggests that simultaneous blockage of endocannabinoid hydrolysis and oxygenation by using the substrate-selective COX-2 inhibitor, which avoids the cardiovascular and gastrointestinal side effects associated with the use of general COX-2 inhibitors, might be a suitable strategy for the treatment of inflammatory and neuropathic pain.

## 1. Introduction

Neuropathic pain is a chronic and debilitating form of pain caused by traumatic nerve injury, toxic insults, and various disease states [1]. The pathogenesis of neuropathic pain is attributed to abnormal synaptic neurotransmission, elevated inflammatory response, and neuronal injury. Although opioids and non-steroidal anti-inflammatory drugs (NSAID) have strong analgesic effects, they are not satisfactory to all the populations, and the severe side effects are major obstacles for their clinical application. Therefore, development of novel therapeutic approaches for neuropathic pain remains an important area of unmet clinical need.

Cannabis has been used for centuries to alleviate pain, but because of its wildly held view for drugs of abuse, development of cannabis-based medicine is severely impeded [2]. Since the discovery of cannabinoid receptors and their endogenous ligands in early 1990s, studies on the therapeutic potential of cannabinoids on pain and other human diseases have increased exponentially [3,4]. Cannabinoids can regulate synaptic transmission, reduce inflammation and neuronal damage in myriad neurological disorders and neurodegenerative diseases, and have been shown to be more effective than opioids in the management of neuropathic pain [5,6]. The endocannabinoid (eCB) system is composed of eCB ligands, their G-protein coupled cannabinoid type 1 and type 2 receptors (CB1 and CB2), and enzymes involved in eCB biosynthesis and degradation. Anandamide (AEA) and 2-arachidonylglycerol (2-AG) are two most studied eCB ligands. AEA is mainly synthesized from N-arachidonoyl phosphatidylethanolamine by N-acyl-phosphatidylethanolamine phospholipase D and degraded by fatty acid amide hydrolase (FAAH) [7]. 2-AG is produced from the membrane phosphatidylinositol-4, 5-bisphosphate by activation of phospholipase C and diacylglycerol lipases and inactivated primarily by monoacylglycerol lipase (MAGL) [8,9]. Unlike the other neurotransmitters, endogenous cannabinoids are not stored in vesicles and are produced where they are needed. This “on demand” synthesis suggests that the production of endocannabinoids is able to activate cannabinoid receptors in a time- and region-specific manner without activating all accessible receptors indiscriminately [7].

Accumulating evidence points to the role of blocking endocannabinoid hydrolysis in the treatment of various pain conditions [10]. However, recent studies suggest that the potency of inhibitors of endocannabinoid hydrolytic enzymes can be reduced by the availability of other metabolic routes, and that may partially explain the lack of efficacy of these inhibitors in the clinical studies [11,12,13]. In addition to inactivation by MAGL and FAAH, 2-AG and AEA can be oxygenated by cyclooxygenase 2 (COX-2) to generate prostaglandin glycerol esters (PG-Gs) and prostaglandin ethanolamides (PG-EAs) [14]. The production of several PG-Gs and PG-EAs has been shown to cause inflammation and excitotoxicity leading to inflammatory and neuropathic pain [15,16]. Thus, the therapeutic efficacy of these endocannabinoids can be diminished by the oxygenated products. It was found that r-enantiomers of ibuprofen, naproxen and flurbiprofen, which were previously considered as inactive COX-2 inhibitors, are the potent substrate-selective COX-2 inhibitors (SSCIs) for endocannabinoids [17]. In fact, r-flurbiprofen has been shown to be a therapeutic agent in the animal model of pain and multiple sclerosis through enhancing the levels of endocannabinoids [17,18,19]. LM4131, the morpholinyl amide of indomethacin, is a novel SSCI that reduced anxiety-like behaviors by selectively increasing the endogenous levels of AEA, without affecting the levels of non-canonical endocannabinoid lipids, such as oleoylethanolamine (OEA) and palmitoylethanolamine (PEA), and the prostaglandin synthesis [20]. Our previous study also showed that systemic repetitive administration of LM4131 significantly reduced inflammatory response in sciatic nerve, dorsal root ganglion and spinal cord, and suppressed hyperalgesia and mechanical allodynia in a mouse model of neuropathic pain induced by chronic constriction injury (CCI) [21]. In addition to enhancing eCB signaling, SSCIs are expected to circumvent the common side effects caused by the traditional non-steroidal anti-inflammatory drugs (NSAIDs), such as cardiovascular risk and gastrointestinal bleeding [17,22]. Despite co-administration of inhibitors for FAAH and COX-2 has shown greater effects to alleviate inflammatory and neuropathic pain [23,24,25], the adverse effects caused by the general COX-2 inhibitors remains a major issue. In this study, we found that the FAAH inhibitor PF04457845 and LM4131 dose dependently reduced thermal hyperalgesia and mechanical allodynia in the mouse CCI model. Combined treatment with sub-effective doses of these two inhibitors greatly attenuated inflammation in both sciatic nerve and spinal cord and was shown to have greater anti-nociceptive effects than the use of each drug alone. This study suggests that combined treatment with the FAAH inhibitor and SSCI might be an ideal strategy for the treatment of neuropathic pain without causing unwanted side effects.

## 2. Materials and Methods

### 2.1. Materials

An FAAH inhibitor, PF04457845, and the substrate-selective COX-2 inhibitor LM4131 were purchased from Cayman Chemicals (Ann Arbor, MI, USA). All other chemicals and reagents were purchased from Sigma (St. Louis, MO, USA) unless stated otherwise.

### 2.2. Animals

Male, 10-week-old C57BL/6J mice were purchased from the Jackson Laboratory (Bar Harbor, ME, USA). Animal care and experimental procedures were carried out in accordance with NIH guidelines and approved by the Uniformed Services University Animal Care and Use Committee.

### 2.3. Chronic Constriction Injury Surgery

The surgical procedure for partial sciatic nerve ligation was carried out as previously described [21,26]. Briefly, after anesthetization of animals with isoflurine (3.5% for induction and 2% for maintenance), the common sciatic nerve was exposed at the mid-thigh level by blunt dissection and separated from the surrounding tissue. Two ligatures of 6-0 sterile silk, spaced 1.0 to 1.5 mm apart, were loosely tied around the sciatic nerve. In the sham-operated mice, the sciatic nerve was dissected and exposed without ligation. The muscles were closed with suture and the skin was closed with staples.

### 2.4. Assessment of Nociceptive Behavior

Nociceptive behavioral tests were performed on day 0 (baseline) and day 7 post-injury 2 h following the drug treatment.

#### 2.4.1. Hot Plate Test

The thermal escape latency was determined using a hot plate test apparatus (Plantar Analgesia Meter, IITC Life Science Inc., Victory Blvd Woodland Hills, CA, USA). Briefly, a metal hot plate surrounded by a transparent glass rectangle was heated to 52 °C. The same groups of animals used for von Frey test were placed individually on the hot plate for 4 trials, separated by at least 15 min. Withdrawal latency was defined as the time required for the animal to shake or lick its hind paw. In the absence of a response at 20 s, the stimulus was terminated, and that latency was assigned.

#### 2.4.2. Von Frey Test

Mechanical allodynia was assessed with the von Frey test, as described earlier by Smits et al. [27]. The level of allodynia was determined by testing the withdrawal response to tactile stimuli with von Frey filament. Mechanical thresholds were determined based on the up-down method [28]. Mice were placed in a Plexiglas cage with mesh metal floor and allowed to acclimate for 30 min before testing. A series of calibrated von Frey filaments (Stoelting, Wood Dale, IL, USA) with logarithmically incremental force ranging from 0.04 g to 2.0 g were applied to the mid plantar surface of the hind paws. Each filament was touched perpendicularly on the paw and held for 3 s. A positive response was noted if the paw was sharply withdrawn or flinching immediately upon removal of the filament. In case of ambiguous response such as ambulation, the stimulus was repeated.

It was previously reported that PF04457845 completely blocked FAAH activity in mouse brain after 1 h intraperitoneal (i.p.) injection and continued for 24 h, and the drug concentrations in brain and plasma were peaked after 4 h oral administration [29]. In line with the biochemical analyses, PF04457845 showed maximal antihyperalgesic effects in the inflammatory pain model when the drug was injected from 2 h to 8 h prior to test [29]. As to LM4131, AEA levels in the brain were increased with LM4131 alone or its combination with PF-3845 after 2 h i.p. injection [20]. After 1 h i.p. injection, animals showed anxiolytic behavior in the open field test at later time period of 1 h session [20]. Based on these findings, we performed the behavioral tests at 2 h following drug treatment.

### 2.5. Drug Treatment

All drugs were dissolved with DMSO as a stock solution and stored at −20 °C until use. DMSO or the drug stock solution was mixed with cremophor EL (Calbiochem, Burlington, MA) and saline with a ratio of 1:1:18. CCI mice were randomly assigned to receive PF04457845, LM4131 or their combination at indicated concentrations, or the vehicle control. Drugs were given intraperitoneally on day 7 post-injury. Animals after behavioral tests were immediately sacrificed for further analysis.

### 2.6. QRT-PCR

The sciatic nerve and lumbar spinal cord were dissected and frozen quickly in dried ice and stored at −80 °C until use. Total RNA was isolated from the frozen tissues using Trizol reagent according to the manufacturer’s protocol (Sigma, St Louis, MO, USA). One µg RNA was reacted with double stranded DNase followed by reverse transcription for cDNA synthesis using MAXIMA cDNA synthesis with dsDNase kit (Thermo Fisher Scientific, Waltham, MA, USA). The qPCR reaction mixture contained LightCycler 480 SYBR Green I PCR master mix (Roche Life Science, Basel, Switzerland), with 20 ng cDNA and 100 nM of each primer. PCR was run according to the manufacturer’s protocol using LightCycler 480 II (Roche Life Sciences). The relative expression levels were determined based on the Ct values of the gene of interest and GAPDH, which was used for normalization. The primer sequences used are shown below: MCP-1 (BC145869): forward, 5’-cagcaagatgtcccaatga-3’ and reverse, 5’-tctggacccattccttcttg-3’; IL-1β (BC011437): forward, 5’-gcaactgttcctgaactcaact-3’ and reverse, 5’-atcttttggggtccgtcaact-3’; Il-6 (GI:124376265): forward, 5’-ccggagaggagacttcacag-3’ and reverse, 5’-cagaattgccattgcacaac-3’ and GAPDH (GU214026): forward, 5’-aggtcggtgtgaacggatttg-3’ and reverse, 5’-tgtagaccatgtagttgaggtca-3’.

### 2.7. Immunostaining

Animals were euthanized by intraperitoneal injection of ketamine and xylazine solution (90 mg ketamine/10 mg xylazine per mL, 10 µL/g body weight), then intracardially perfused with ice cold PBS followed by 4% paraformaldehyde in the same buffer. Spinal cords were dissected out and kept in 4% paraformaldehyde at 4 °C overnight. Tissues were then transferred into PBS containing 30% sucrose at 4 °C overnight and stored at −80 °C after being embedded in Tissue Tek OCT. Transverse sections of lumbar spinal cords were cut at 14 μm using a cryostat (Leica model CM1950, Bannockburn, IL, USA) and mounted onto Superfrost Plus slides for immunoassay. Rat anti-Iba1 (1:300; Abcam, Cambridge, MA, USA) and mouse anti-GFAP (1:500; Cell Signaling, Danvers, MA, USA) antibodies were used for immunofluorescence assay. Briefly, the slides were washed with PBS twice, and then incubated in PBS containing 1% donkey serum and 0.3% Triton X-100 for 30 min at room temperature, followed by respective primary antibody incubation in the same buffer overnight. The slides were then washed with PBS containing 0.2% Triton X-100 and incubated with Alexa fluor 488 or Alexa fluor 594 conjugated donkey anti-mouse or rat secondary antibodies (1:750; Thermo Fisher Scientific, Waltham, MA, USA) for 1 h. The slides were again washed with PBS twice and covered with Fluoroshield mounting medium with DAPI. Immunofluorescence images were obtained with a fluorescence microscope (Nikon Eclipse TE-2000 U). All immunofluorescence data were obtained in a minimum of 5–7 serial sections from the lumbar spinal cord of the same animal. Iba1 and GFAP immunoreactivities were quantified by counting immuno-positive cells and results were given as mean cell numbers per mm^2^. Immunostaining without the primary antibodies was carried out for negative controls.

### 2.8. ELISA Assay

Protein levels of IL-6 and MCP-1 were measured by sandwich enzyme linked immunoassay (ELISA). Briefly, the tissue cell lysate was prepared with RIPA buffer [10 mM Tris-HCl pH 7.4, 30 mM NaCl, 1 mM EDTA, 1% Nonidet P-40, supplemented with 1 mM Na_3_VO_4_ and complete Mini protease inhibitor cocktail (Roche Life Science)] from fresh ipsilateral sciatic nerves and spinal cords. The ELISA for IL-6 and MCP-1 were performed according to the manufacturer’s protocol (Thermo Fisher, Waltham, MA, USA). For PGE_2_ assay, mouse sciatic nerve isolated at day 7 post-injury was homogenized with 40 µL of 0.02% trifluoroacetic acid and 100 µL of acetonitrile on ice. The homogenate was then further dispersed in 1 mL of acetonitrile by vortex and left at 4 °C overnight. On the next day the supernatant after centrifugation at 2000 g for 5 min to remove the debris was evaporated, then reconstituted with EIA buffer supplemented in the PGE_2_ EIA kit (Cayman Chemical, Ann Arbor, MI, USA). EIA was performed following the manufacturer’s protocol.

### 2.9. Statistical Analysis

Data were analyzed with one-way analysis of variance (ANOVA) for biochemical and immunohistological data, and two-way ANOVA for behavioral tests. Dunnett’s multiple comparisons test was conducted comparing to the CCI/vehicle group. Results were represented as mean ± SD. F value and *p* value were calculated using GraphPad Prism 9.4.1. software. A significant difference was determined as *p* < 0.05, and *p* value is denoted as asterisks, *, *p* < 0.05; **, *p* < 0.01, ***, *p* < 0.001, ****, *p* < 0.0001. Total used animal number was 111.

## 3. Results

### 3.1. PF04457845 Dose-Dependently Attenuated CCI-Induced Thermal Hyperalgesia and Mechanical Allodynia

**Figure 1:**
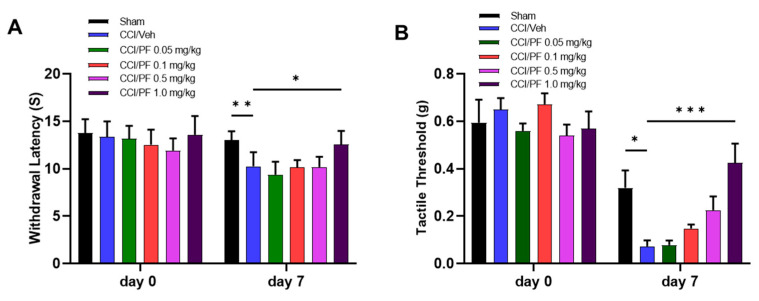
PF04457845 dose dependently alleviated neuropathic pain in the CCI mouse. At days 0 and 7 after surgery, thermal withdrawal latency was determined by the hot plate test (**A**) and mechanical thresholds were measured by von Frey test using the up-down method (**B**) 2 h post injection. On day 7 post-injury, CCI/vehicle group significantly reduced withdrawal latency compared to the sham. Treatment with PF04457845 at 1.0 mg/kg but not at 0.05, 0.1 and 0.5 mg/kg showed a significantly increased thermal withdrawal latency (**A**). Mechanical threshold was increased by PF04457845 dose-dependently, but a significant reduction was observed at 1.0 mg/kg (**B**). *n* = 5 to 7/group. F (5, 30) = 2.954 in (**A**) and F (5, 29) = 5.234 in (**B**). *, *p* < 0.05; **, *p* < 0.01, ***, *p* < 0.001.

To determine the dose-dependent effects of the selective FAAH inhibitor PF04457845 in the animal model of neuropathic pain, CCI mice were randomly assigned and given intraperitoneal injection of four different doses of PF04457845 (0.05, 0.1, 0.5, and 1.0 mg/kg) or vehicle on day 7 post-injury, and the hot plate and von Frey tests were conducted 2 h later. Compared to sham control animals, the CCI animals showed a significant reduction in thermal withdrawal latency (Figure 1A) and increase in hind paw tactile sensitivity by reduced tactile threshold (Figure 1B). The group treated with PF04457845 at 0.05 mg/kg and 0.1 mg/kg showed no differences from the CCI/vehicle group in the hot plate test and the von Frey test. However, animals treated with 1 mg/kg PF04457845 completely blocked both thermal hyperalgesia and mechanical allodynia in the CCI mice. The withdrawal latency and the tactile threshold were 12.6 s and 0.43 g, respectively, and those values were similar to the withdrawal latency of 13.0 s and tactile threshold of 0.32 g in the sham animals.

### 3.2. Concentration Dependent Effect of LM4131 in the CCI Induced Mechanical Allodynia and Thermal Hyperalgesia

**Figure 2:**
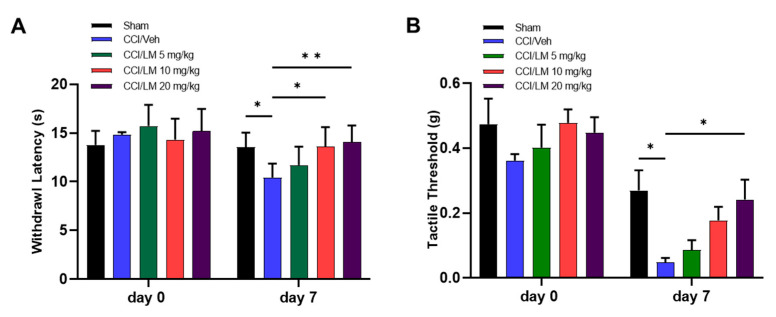
CCI induced mechanical allodynia, and thermal hyperalgesia were attenuated by LM4131 dose-dependently. Experimental procedures were followed by Figure 1. Treatment with LM-4131 (LM) at 10 and 20 mg/kg, but not at 5 mg/kg, ameliorated thermal sensitivity (**A**), and the hyperalgesia was significantly reduced by LM at 20 mg/kg (B). *n* = 5-7/group. F (4, 20) = 6.128 in (**A**) and F (4, 20) = 1.866 in (**B**). *, *p* < 0.05; **, *p* < 0.01.

To determine the concentration dependent effect of the substrate-selective COX-2 inhibitor LM4131, three different doses of LM4131 (5, 10 and 20 mg/kg) were given to the CCI mice 7 days post-injury. Although treatment with 5 mg/kg and 10 mg/kg of LM4131 showed partial effects, 20 mg/kg administration significantly attenuated both mechanical allodynia and thermal hyperalgesia. Animals treated with 10 and 20 mg/kg of LM4131 had longer endurance to the thermal stimulus with withdrawal latency of 13.6 s and 14.0 s that were significantly higher than the value in the CCI/vehicle group (10.4 s) (Figure 2A). The tactile threshold of the 20 mg/kg treated groups was 0.24 g, which was significantly greater than 0.048 g in the CCI/vehicle group.

### 3.3. Co-Treatment with Subeffective Doses of LM4131 and PF04457845 Suppressed Neuropathic Pain

**Figure 3:**
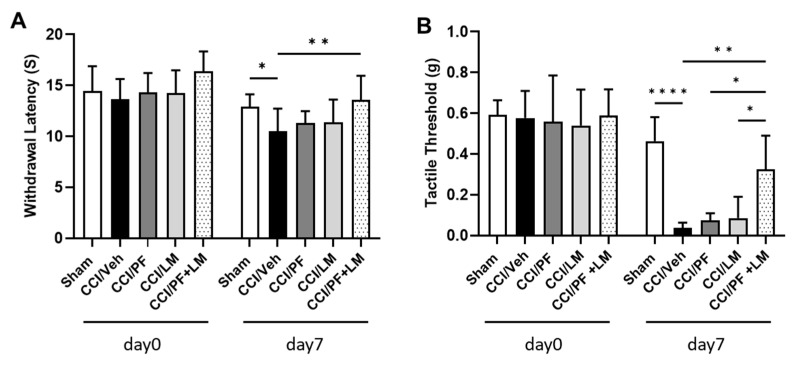
Treatment with LM4131 and PF04457845 synergistically suppressed hyperalgesia induced by CCI. On 7 days post-injury, the CCI mice were treated either with LM (5 mg/kg), PF (0.5 mg/kg) alone or their combination. Neuropathic pain behavior was assessed at days 0 and 7 post-injury by hot plate (**A**) and von Frey tests (**B**). As shown in (**A**,**B**), individual treatment with low dose of LM or PF had no therapeutic effect on the CCI mice compared to the CCI/vehicle groups. The combination of low dose of LM and PF greatly attenuated pain hypersensitivity, indicated by prolonged withdrawal latency and elevated tactile threshold. F (4, 44) = 0.9180, *n* = 10/group in (**A**) and F (4, 23) = 6.222, *n* = 5 to 7/group in (**B**). *, *p* < 0.05; **, *p* < 0.01, ****, *p* < 0.0001.

To determine whether treatment with LM4131 and PF04457845 has an enhanced antinociceptive effect, CCI animals were treated with sub-effective doses of either PF04457845 (0.5 mg/kg) or LM4131 (5 mg/kg) or their combination on day 7 post-injury (Figure 3A,B). As expected, neither PF04457845 nor LM4131 alone showed any significant difference from the vehicle group; however, the drug combination exhibited significant improvements in both the hot plate and the von Frey test. The average withdrawal latency in the combination treatment group was 13.6 s, whereas those of the vehicle, PF04457845 and LM4131 treatment groups were 10.5 s, 11.3 s, and 11.4 s, respectively (Figure 3A). Similarly, the combination treatment group showed an increase in tactile threshold (0.33 g), while the tactile thresholds in the PF04457845 and LM4131 alone treatment groups were 0.07 g and 0.09 g, respectively (Figure 3B). These results suggested that although the use of low sub-effective dose of LM4131 (5 mg/kg) or PF04457845 (0.5 mg/kg) was unable to suppress neuropathic pain, CCI induced hyperalgesia was significantly blocked by co-administration of these compounds. Our data indicated that combined treatment with selective inhibition of endocannabinoid oxygenation and inhibition of the AEA hydrolysis attenuated neuropathic pain in CCI animals effectively.

### 3.4. Combination of PF04457845 and LM4131 Suppressed Inflammatory Response in Sciatic Nerve and Spinal Cord

**Figure 4:**
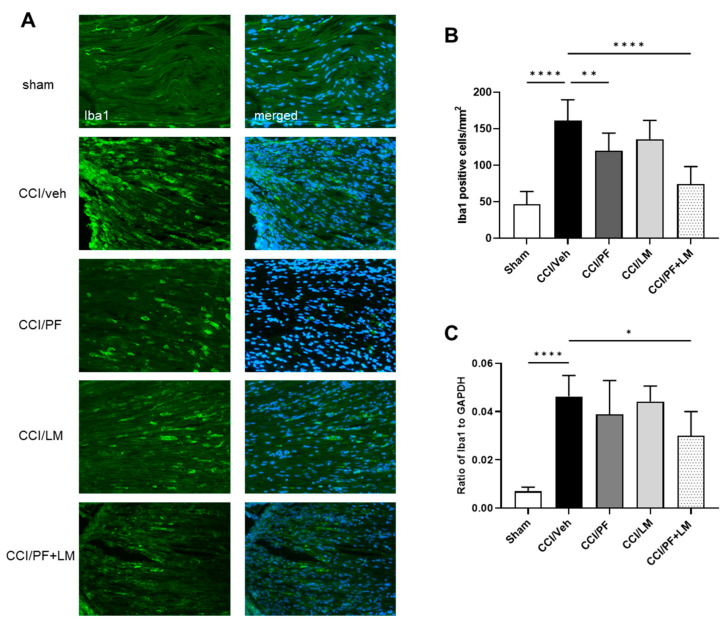
Macrophage infiltration in the ipsilateral sciatic nerve was attenuated by co-treatment with LM4131 and PF04457845. Representative Iba1 positive macrophage images were shown in the left panels and merged with DAPI staining in the right panels (**A**). The number of macrophages in the combined LM and PF treatment group was reduced compared to that in the CCI/vehicle group (**A**,**B**). The increased levels of Iba1 mRNA in the sciatic nerve were also significantly reduced by the combination, but not by each drug alone (**C**). F (4, 35) = 29.35 in ((**B**); *n* = 8/group) and F (4, 19) = 15.59 in ((**C**); *n* = 4 to 5/group). *, *p* < 0.05; **, *p* < 0.01, ****, *p* < 0.0001.

**Figure 5:**
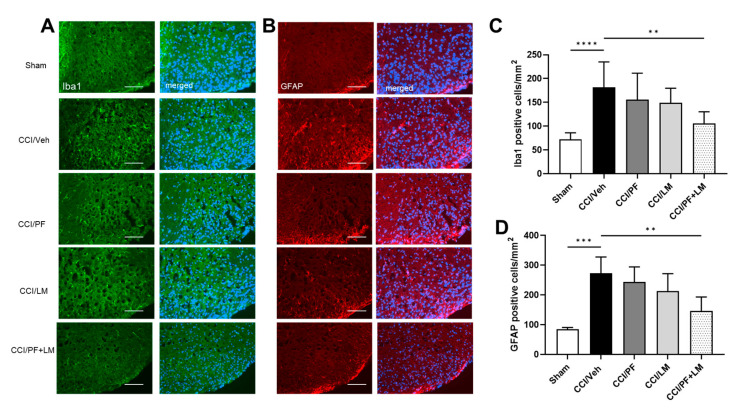
Combination of LM4131 and PF04457845 attenuated microglia and astrocyte activation in the CCI mouse spinal cord. Panel A shows representative anti-Iba1 immunofluorescent images (left panels) and merged images with DAPI staining (right panels). Panel B shows anti-GFAP immunofluorescent images (left panels) and merged images with DAPI staining (right panels). Iba1 positive microglia/macrophages (**A**) and GFAP positive activated astrocytes (**B**) were reversed by the co-treatment. Quantitative analyses based on A and B were shown for microglia/macrophage (**C**) and activated astrocyte (**D**). F (4, 35) = 9.771 in ((**C**); *n* = 8/group) and F (4, 15) = 10.42 in ((**D**); *n* = 5/group). **, *p* < 0.01, ***, *p* < 0.001, ****, *p* < 0.0001.

Activation of microglia and astrocytes in the spinal cord dorsal horn and infiltration of macrophages to the peripheral nerve perform a pivotal role in the initiation and development of neuropathic pain [30]. In this study, we assessed low doses of PF04457845 and LM4131 co-administration on macrophage accumulation in the CCI mouse sciatic nerve. At 7 days post-injury, increase in macrophage infiltration in the CCI mouse sciatic nerve was observed, since the number of Iba1 positive cells was more than 3-fold increased (Figure 4B). Although Iba1 positive cells in the PF04457845 or LM4131 group showed a moderate reduction compared to the vehicle group (119 ± 8 cells/mm^2^ and 136 ± 10 cells/mm^2^, respectively), the combined PF04457845 and LM4131 treatment group showed a distinct reduction (75 ± 8 cells/mm^2^) (Figure 4B). Consistently, the mRNA expression of Iba1 was also significantly increased in the CCI/vehicle group. Combined drug treatment, but not either PF04457845 or LM4131 alone reduced Iba1 gene expression (Figure 4C). In addition to suppressed macrophage accumulation in the sciatic nerve, combined low doses of PF04457845 and LM4131 treatment also attenuated microglia and astrocyte activation in the CCI mouse spinal cord (Figure 5). Based on Iba1 and GFAP immunopositive cells, there was a partial reduction in the numbers of microglia and activated astrocytes in the spinal cord either by low dose of PF04457845 or LM4131. Only the combination of PF04457845 and LM4131 significantly reversed the number of Iba1 positive cells (106 ± 9 cells/mm^2^), which was increased by CCI (181 ± 19 cells/mm^2^) (Figure 5A,C), and that of GFAP positive cells that were 146 ± 24 cells/mm^2^ compared to 273 ± 27 cells/mm^2^ in CCI/veh group (Figure 5B,D).

### 3.5. PF04457845 and LM4131 Co-Treatment Attenuated the Inflammatory Cytokines/Chemokines Levels in the Sciatic Nerve and Spinal Cord

**Figure 6:**
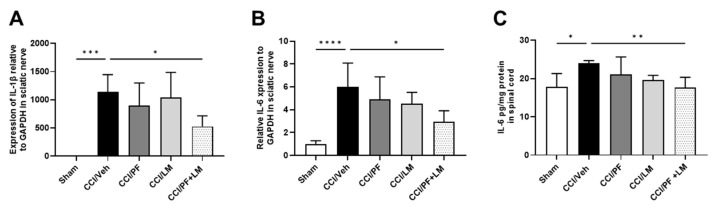
Inflammatory cytokine expression was attenuated in sciatic nerve and spinal cord by co-treatment of LM4131 and PF04457845. Real-time PCRs were performed to determine the relative mRNA expression of IL-1β (**A**) and IL-6 (**B**) in sciatic nerve. IL-6 production in the spinal cord was determined by ELISA (**C**). *n* = 4 to 5/group. F (4, 19) = 9.189 in (**A**), F (4, 20) = 9.050 in (**B**), and F (4, 18) = 3.851 in (**C**). *, *p* < 0.05; **, *p* < 0.01, ***, *p* < 0.001, ****, *p* < 0.0001.

The increased immune cell infiltration in the CCI mouse sciatic nerve would contribute to the development of neuropathic pain by secreting pro-inflammatory cytokines such as TNFα, IL-1β and IL-6. In the previous study we have demonstrated that chronic treatment with LM4131 protects against neuropathic pain by suppressing inflammatory cells accumulation and pro-inflammatory cytokines production in the CCI mouse model [21]. Increase in expression of IL-1β and IL-6 mRNA in the sciatic nerve and IL-6 protein production in the ipsilateral spinal cord of the CCI mice on 7 days post injury (Figure 6). Either 0.5 mg/kg of PF04457845 or 5 mg/kg of LM4131 slightly downregulated levels of IL-1β and IL-6 mRNAs and IL-6 protein compared to the CCI/vehicle group. Co-administration of PF04457845 and LM4131 significantly reduced those genes (Figure 6A,B) and the protein (Figure 6C).

**Figure 7:**
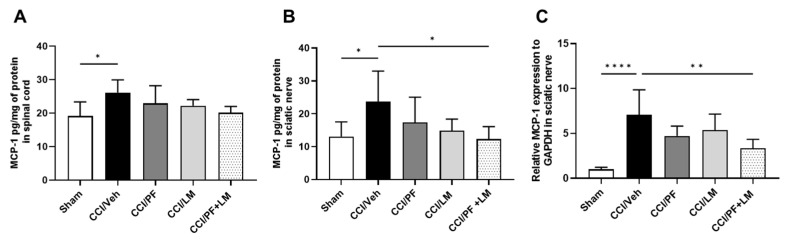
Elevated MCP-1 in the sciatic nerve and spinal cord of the CCI were suppressed by LM4131 and PF04457845 combined treatment. Tissue lysates from sciatic nerve (**A**) and spinal cord (**B**) at 7 days post-injury were subjected to the MCP-1 ELISA. The increased protein of MCP-1 in the CCI/vehicle group was significantly suppressed by co-administered with the low doses of two inhibitors. Total RNA from sciatic nerve was subjected to qRT-PCR. Increased MCP-1 expression in CCI mice was reversed by combined treatment group (**C**). *n* = 4 to 5/group. F (4, 18) = 2.518 in (**A**), F (4, 19) = 2.575 in (**B**), and F (4, 18) = 9.504 in (**C**). *, *p* < 0.05; **, *p* < 0.01, ****, *p* < 0.0001.

**Figure 8:**
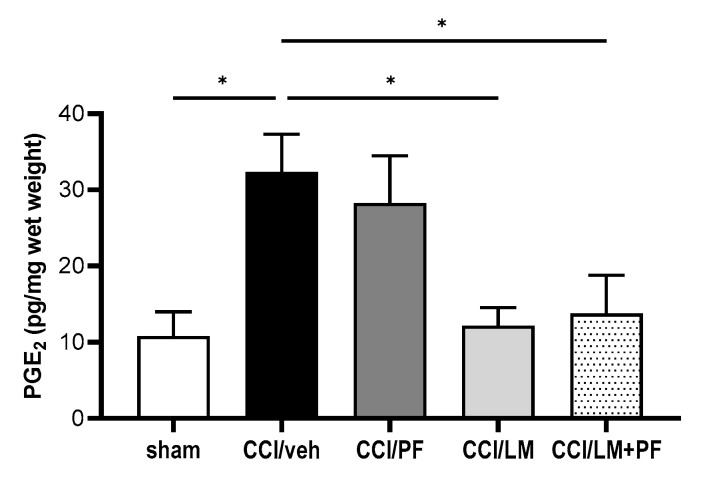
LM4131 and PF04457845 co-treatment reduced PGE_2_ synthesis in the CCI mouse sciatic nerve. The sciatic nerve tissues from mice 7 days post-injury were subjected to PGE_2_ ELISA. Treatment with LM4131 and combined treatment significantly reduced PGE_2_ production. F (4, 14) = 4.599, and *n* = 3 to 4/group. *, *p* < 0.05.

Next, we examined the mRNA and protein expression of MCP-1, which is crucial for the development of neuropathic pain in both sciatic nerve and spinal cord at 7 days post-CCI. The protein levels of MCP-1 in the ipsilateral sciatic nerve and spinal cord were measured by enzyme immunoassay (Figure 7A,B). The production of MCP-1 in both regions significantly increased in the CCI/vehicle group compared to the sham group. In both tissues, either PF04457845 or LM4131 alone partially reduced the production of MCP-1 compared to the CCI/vehicle group. However, co-administration of these inhibitors suppressed MCP-1 comparable to the level in the sham group (Figure 7A,B), although co-treatment did not reduce in the spinal cord significantly (Figure 7A). Combined treatment with PF04457845 and LM4131 also reduced the mRNA expression of MCP-1 in the CCI mouse sciatic nerve (Figure 7C). PGE_2_ is the important pro-inflammatory and critical nociceptive mediator in neuropathic pain by the sciatic nerve injury. Our previous studies showed a significant increase in PGE_2_ in the CCI model [21,26]. To further examine the mechanism attributing to the therapeutic effect of the combination of PF04457845 and LM4131, we conducted ELISA of PGE_2_ in the ipsilateral side on day 7 post-injury (Figure 8). PGE_2_ production was increased 3-fold in the CCI/vehicle group. While reduction by PF04457845 alone was partial, LM4131 treatment and their combination showed a significant reduction, suggesting that the reduction in PGE_2_ was mainly attributed by LM4131.

## 4. Discussion

Numerous studies have suggested that chronic neuropathic pain resulting from peripheral nerve damage is a world-wide clinical issue, therefore, development of novel therapeutic approaches is an area of unmet clinical needs. Cannabinoids have been shown to regulate synaptic transmission, reduce inflammation and neuronal damage in a number of neurological disorders and neurodegenerative diseases, and are thought to be more effective than opioids in the management of neuropathic pain [2,5,31,32,33]. Although augmentation of endocannabinoids 2-AG and AEA by inhibiting their hydrolytic enzymes is shown to alleviate both inflammatory and neuropathic pain [1,34] the previous studies indicate that inhibition of 2-AG and AEA hydrolysis can facilitate their oxygenation by COX-2 and produce PG-Gs and PG-EAs causing inflammation and excitotoxicity [13,35]. Thus, blocking both endocannabinoid hydrolysis and oxygenation is believed to be more clinically useful [13,36]. Our current study showed that co-treatment with lower doses of the AEA hydrolysis inhibitor PF04457845 and the substrate-selective COX-2 inhibitor LM4131, which is expected to be devoid of the cardiovascular and gastrointestinal side effects resulting from the use of classical cyclooxygenase inhibitors, significantly reduced hyperalgesia and allodynia, and attenuated inflammatory response in the CCI mouse sciatic nerve and spinal cord.

AEA and 2-AG, two dominant endocannabinoids, are present in key regions involved in the detection, relay and integration of nociceptive inputs, that include skin, DRG, spinal cord dorsal horn, periaqueductal gray (PAG) and rostral ventromedial medulla [33,37]. Previous studies have shown that following the induction of inflammatory and neuropathic pain, cannabinoid receptors and their endogenous ligands AEA and 2-AG are elevated in various regions of the pain pathway [38,39], suggesting that enhancement of endocannabinoid signaling might combat pain. Consistent with the studies of other FAAH inhibitors [40,41,42], we found that PF04457845 attenuated neuropathic pain in the CCI mouse model. Administration of PF04457845 at 1.0 mg/kg resulted in a complete inhibition of neuropathic pain, demonstrating increased withdrawal latency and tactile threshold. The similar results were also found in the CCI mice treated with LM4131 in a dose dependent manner. Though there was less therapeutic effect with lower doses of either PF04457845 (0.5 mg/kg) or LM4131 (5 mg/kg) alone, their combination possessed an enhanced therapeutic effect in the mouse CCI model.

The role of glial activation and cytokine production in neuropathic pain has been extensively studied [43,44,45]. Despite microglia comprise less than 20% of spinal glial cells in physiological conditions, microglia and the infiltrated macrophages can accumulate profoundly in the DRG and spinal cord after nerve injury [43]. Upon activation, microglial cells stimulate the complement components of the immune system and release cytokines, chemokines, and other cytotoxic substances such as nitric oxide and free radicals to facilitate the onset and development of neuropathic pain [44,46,47]. In this study, we found that co-administration of lower doses of PF04457845 and LM4131 significantly reduced the number of Iba1 positive cells, and the mRNA expression. This phenomenon was not observed when the same dose of PF04457845 or LM4131 was individually administered. These results suggested that dual inhibition of AEA hydrolysis and oxygenation is more potent to suppress inflammation and neuropathic pain triggered by these inflammatory cells.

Growing evidence supports that cytokines act as modulators of neuronal plasticity and promote nociceptive transmission under neuropathic pain conditions. IL-1β is one of the first cytokines to be implicated in peripheral nerve injury-induced neuropathic pain [46,48]. IL-1β acts on the sensory neurons in the dorsal horn and the central terminals of primary afferents to activate signaling pathways in immune cells initiating neuropathic pain. Genetic deletion of both IL-1β and IL-1α showed a remarkable reduction in mechanical hypersensitivity in mouse models of peripheral nerve injury and CCI of the sciatic nerve [48]. Il-6, another well studied cytokine, also performs a crucial role in neuropathic pain caused by peripheral nerve injury, spinal cord damage, and chemotherapy-induced peripheral neuropathy [49]. The involvement of IL-6 was also demonstrated in peripheral neuropathy in animal models of sciatic cryoneurolysis and CCI of the sciatic nerve [50,51]. IL-6 mRNA was significantly elevated in both dorsal and ventral horns in neuropathic pain models of spinal nerve cryoneurolysis and spinal nerve tight ligations [52]. Ramer et al. reported that spinal nerve lesion-induced mechanical allodynia was attenuated in IL-6 knockout mice of sciatic cryoneurolysis [53]. Moreover, an intrathecal injection of anti-IL-6 antibody was found to attenuate L5 spinal nerve transection induced mechanical allodynia [52]. All these results support the important role of central IL-6 in the etiology of mechanical allodynia following spinal nerve and peripheral nerve injury. Given that IL-1β and Il-6 are two indispensable nociceptive molecules, the expression levels of IL-1β and Il-6 from sciatic nerve and spinal cord were measured to evaluate the combined drug treatment in our CCI animal model. The expression of IL-1β and Il-6 was significantly increased in the ipsilateral sciatic nerve at 7 days post-injury. Despite the use of lower doses of LM4131 or PF04457845 was unable to suppress inflammatory response, the increased production of IL-1β and Il-6 in the ipsilateral sciatic nerve of the CCI/vehicle group was remarkably reduced by combined treatment with these inhibitors at sub-effective doses. This result further supports that combination of low doses of LM4131 and PF04457845 may exert a synergistic effect to alleviate neuropathic pain.

In addition to pro-inflammatory cytokines, chemokines are also known to perform crucial roles in the development of neuropathic pain. MCP-1 has been proposed to be an important molecule promoting nociceptive transmission after peripheral injury [54]. The injury induced expression of MCP-1 in DRG neurons has been demonstrated under many neuropathic pain conditions [55,56]. Increased MCP-1 was shown to result in a leakage of the blood-spinal cord barrier and enable the infiltration of peripheral immune cells into the spinal cord [54,57]. It is known that macrophages and T lymphocytes contribute to pain hypersensitivity. Increased production of MCP-1 not only activates microglia but also sensitizes dorsal horn neurons to the noxious stimuli. To investigate if combination of low doses of PF04457845 and LM4131 treatment could modulate MCP-1 production in the CCI mouse model, both the mRNA and protein levels of MCP-1 were examined. In the ipsilateral sciatic nerve and spinal cord, significantly increased MCP-1 production was detected in the CCI/vehicle group. Despite low doses of PF04457845 or LM4131 could not block the elevated levels of MCP-1, a significant reduction of MCP-1 was seen in sciatic nerve when low doses of LM-4131 and PF04457845 were co-administered. These findings indicate that combination of low dose of PF04457845 and LM4131 is likely to affect neuropathic pain initiation and progression via blocking the production of cytokines and chemokines.

Combined treatment with the AEA hydrolysis and oxygenation inhibitors has been shown to exert greater antinociceptive effects [23,24]. The endocannabinoids AEA and 2-AG are metabolized by the canonical hydrolysis pathway, and the alternative oxygenation pathway catalyzed by COX-2 [17,22]. Among the oxygenation products, some species of PG-Gs and PG-EAs have been shown to cause inflammation, excitotoxicity, and inflammatory and neuropathic pain [10,58]. Inhibition of COX-2 not only increases the endogenous levels of AEA and 2-AG, but also blocks the production of the inflammatory oxidation products. Several studies have found that LM4131 is a novel SSCI that reduces anxiety-like behaviors by selectively increasing the endogenous levels of AEA, without affecting the levels of non-canonical endocannabinoid lipids, such as OEA and PEA, and the prostaglandin synthesis [22,59]. Our recent study showed that daily treatment with LM4131 significantly reduced inflammatory response in sciatic nerve, DRG and dorsal spinal cord, and suppressed hyperalgesia and mechanical allodynia in a mouse model of CCI induced neuropathic pain [21]. To increase their potential of clinical application, dual inhibition of endocannabinoid hydrolysis and oxygenation would be beneficial by using minimal doses of these inhibitors. The discovery and development of SSCI made it possible to achieve the therapeutic effect while mitigating the adverse effects of the canonical COX inhibitors. Our data showed that the increased PGE_2_ in the CCI sciatic nerve was significantly reduced by the dual drug treatment which was predominantly mediated by LM4131. Suppression of PGE_2_ levels by PF04457845 was minimal, possibly because PGE_2_ production derived from endogenous AEA hydrolysis is not substantial due to the lower concentration of AEA compared to that of 2-AG. Thus, more investigation is needed to develop novel SSCIs that possess high potency and selectivity to the endocannabinoids, but not COX-2 enzymes.

In conclusion, we have found that combination of the subthreshold doses of PF04457845 and LM4131 can effectively ameliorate thermal hyperalgesia and mechanical allodynia through reduction in the inflammatory cell infiltrates, proinflammatory cytokines production and PGE_2_ synthesis in the injured sciatic nerve and ipsilateral spinal cord. This study suggests that manipulation of both endocannabinoid and eicosanoid metabolic pathways through the use of endocannabinoid hydrolysis inhibitors and SSCI is likely to be more clinically useful for the pain treatment.

## Data Availability

The data presented in this study will be made available upon reasonable request.
